# The Epidemiology, Transmission, Genotypes, Replication, Serologic and Nucleic Acid Testing, Immunotolerance, and Reactivation of Hepatitis B Virus

**DOI:** 10.1016/j.gastha.2023.10.008

**Published:** 2023-10-24

**Authors:** Ankoor Patel, Zahra Dossaji, Kapil Gupta, Katerina Roma, Toni-Marie Chandler, Carlos D. Minacapelli, Kaitlyn Catalano, Robert Gish, Vinod Rustgi

**Affiliations:** 1 Internal Medicine, Robert Wood Johnson Medical School, Rutgers Biomedical and Health Sciences (RBHS), Rutgers University, New Brunswick, New Jersey; 2 Division of Gastroenterology and Hepatology, Rutgers Robert Wood Johnson Medical School, New Brunswick, New Jersey; 3 Internal Medicine, Kirk Kerkorian School of Medicine at the University of Nevada, Las Vegas, Nevada; 4 Center for Liver Diseases and Masses, Rutgers Robert Wood Johnson Medical School, New Brunswick, New Jersey; 5 Hepatitis B Foundation, Doylestown, Pennsylvania

**Keywords:** Hepatitis B Virus, Epidemiology of HBV, HBV genotype, HBV eeactivation, Immunotolerance

## Abstract

The epidemiology of Hepatitis B virus (HBV) has drastically changed in recent decades due to public health initiatives, including universal infant vaccination programs,urbanization driving global travel, and migration patterns. Despite screening of pregnant women and newborns significantly reducing the rate of perinatal transmission in certain parts of the world, other, perhaps more uncommon, routes (e.g., parenteral) have led to outbreaks in specific areas affected by the opioid epidemic and injection drug use. Although our current understanding of the effect of genetic variants of HBV is lacking, we review current knowledge and patterns of genetic variants with geographical predominance, pathophysiology, and clinical manifestations. Serologic and molecular markers are used to screen, identify phase and activity of infection, and monitor response to antivirals and/or reactivation. This review will provide the most up-to-date summary of the epidemiology, transmission, genotype, replication, and current methods of screening to follow the various phases of HBV, including immunotolerance and reactivation.

## Introduction

Viral hepatitis (A, B, C, and E) represents the seventh leading cause of worldwide mortality.^1^ Hepatitis B virus (HBV) infection is a global public health issue and a major cause of chronic hepatitis, cirrhosis, and hepatocellular carcinoma (HCC). HBV accounts for up to 42% of patients with cirrhosis and up to 56% of patients with HCC.^2^ The epidemiology of HBV has changed over time and will continue to evolve due to several factors, including vaccination and migration patterns affecting the transmission of the virus globally. The clinical course of HBV can be highly variable and differs between acute and chronic infection. The phases of the disease are differentiated and monitored via serological and nucleic acid-based tests. Over the last several decades, better understanding of the virus has led to advances in antiviral therapies, such as pegylated interferon (PegIFN) and nucleos(t)ide analogues (NAs). However, these advances have been limited with our current antiviral agents; the need still exists for better medications that can lead to functional, complete, and sterilizing “cures.”

## Epidemiology

### Global Epidemiology

Chronic HBV infection has a global prevalence estimated to be between 257 and 291 million affected individuals; the Polaris group provides the current best estimate of 262 million, derived from a large merged database of various serosurveys.^3^ Approximately 15%–40% of people with chronic HBV (CHB) will develop liver cirrhosis, liver failure, or HCC with a 15%–25% estimated rate of mortality.^4^^,^^5^ In the 2010 Global Burden of Disease Study, HBV was estimated to have resulted in 786,000 deaths, with 341,000 deaths attributed to liver cancer and 312,000 deaths attributed to cirrhosis. As a result, HBV infection was ranked fifteenth among all causes of human mortality.^6^^,^^7^ In 2019, the World Health Organization (WHO) estimated mortality from CHB accounted for approximately 820,000 deaths.^8^ In the Risk Evaluation of Viral Load Elevation and Associated Liver Disease/Cancer in HBV (REVEAL-HBV) study, a 11-year, population-based study including 3582 Taiwanese patients followed every 6-12 months with routine ultrasound examinations, the cumulative incidence of cirrhosis was directly correlates with HBV-DNA levels. The highest incidence of cirrhosis was associated with HBV-DNA levels ≥10^6^ copies/ml (200,00 IU/mL) at enrollment. Similarly, the risk of HCC was directly correlated with HBV-DNA levels independent of HBeAg, serum alanine aminotransferase (ALT), and cirrhosis.^9^, ^10^, ^11^, ^12^

The prevalence of HBV infection varies depending on geographic areas and specific populations and is generally higher among males.^13^ Over the last several decades, the epidemiology of HBV has changed due to the impact of universal infant vaccination programs, and through migration between high- and low-prevalence populations.^14^ The prevalence of HBV, which is defined as hepatitis B surface antigen (HBsAg) positivity, is classified into 4 levels: low (<2 %), lower intermediate (2%–4.9%), higher intermediate (5%–7.9%), and high (≥8 %) endemicity rates.^15^ It is estimated that 45%–60% of the global population live in areas of high endemicity.^16^, ^17^, ^18^ Areas where HBV is highly endemic include parts of Asia, sub-Saharan Africa, the Pacific, parts of the Amazon Basin, parts of the Middle East, the central Asian Republics, the Indian subcontinent, and some countries of Central and Eastern Europe.^19^

In areas with high prevalence of HBV, the virus is commonly transmitted via perinatal transmission or horizontal transmission (possibly from vectors other than other children). Ninety percent of the population in these areas has demonstrated serologic exposure at some point in time.^20^ Regions of high endemicity also have high rates of HCC. HCC is one of the 3 major causes of cancer death in Asia, the Pacific Rim, and sub-Saharan Africa.^21^ Early implementation of universal infant vaccination has made tremendous impact in previously high prevalence areas such as China and Gambia. In China, the prevalence of HBsAg decreased from 9.7% to 1.0% in children younger than 5 years between 1992 and 2006, preventing approximately 16 to 20 million cases of CHB.^22^ In addition, the protective efficacy of infant vaccination in preventing CHB infection was reported as 95% according to the Gambia Hepatitis Intervention Study.^23^ A reduction in the incidence of HCC in these populations mirrored the significant reduction in CHB due to vaccination implementation.

Regions of the world that are classified as intermediate-endemic zones include North Africa, the Middle East, parts of Eastern and Southern Europe, South America, and South Asia. These areas contain a similar proportion of the global population as the high prevalence areas mentioned above (> 40%).^24^ Similar to high prevalence areas, perinatal and horizontal transmission are most common.^25^ Vaccination efforts have led to decreases in prevalence in intermediate-prevalence countries in Europe, such as Spain and Italy.^26^, ^27^, ^28^ Compared to high- and intermediate-prevalence regions, low-HBV-prevalence areas make up the minority, or approximately 12% of the global population. Low-prevalence regions include Australia, Asia, Northern and Western Europe, Japan, North America, and some countries in South America.^14^ Most infections are transmitted during adolescence and adulthood via sexual contact, intravenous drug use (IVDU), and other blood-related exposures, including in health care settings.^14^ Areas of low-HBV-prevalence with a relatively high number of people who inject drugs (PWIDs) include Eastern Europe (280,000; 22.8% of the global HBsAg-positive IVDU population) and North America (272,500; 22.2% of the global HBsAg-positive IVDU population).^29^ Other populations which are more likely to acquire HBV in adulthood include incarcerated adults, men who have sex with men, sex workers, and those who are homeless.^30^

Global migration between higher-prevalence countries to lower-prevalence countries is another determinant of CHB burden. In a community-based screening program in New York City, Chinese-born individuals and specifically those born in the Fujian province had the highest seroprevalence (23.2% and 33.1%, respectively).^31^ Similarly, sub-Saharan origin was independently associated with ongoing or past HBV infection in a study conducted in southern Italy.^32^ In lower-prevalence countries, most people with CHB are migrants from endemic areas. In several low-endemic countries in Europe (e.g., Italy, Germany), higher HBV prevalence rates have been observed in migrants and refugees originating from outside Europe compared to the native population. The prevalence of HBsAg chronic carriers in the general population decreased to nearly 1% by 2010 in Italy. Of the 926 migrants or refugees screened, 9% were HBsAg-positive, with the highest HBsAg positivity rate in individuals from Saharan Africa (12%), followed by Eastern Europe (6%) and Northern Africa (2%).^28^^,^^33^^,^^34^

### US Epidemiology

According to the Centers for Disease Control and Prevention (CDC), an estimated 862,000 individuals had CHB infection in the United States in 2016.^8^ In 2018, a total of 3322 acute hepatitis B cases were reported to the CDC with an estimated number of new infections being closer to 21,600 infections after accounting for under-ascertainment and under-reporting. The reported case count correlated to a rate of 1.0 cases per 100,000 population.^35^ A systematic review and meta-analysis by Wong et al in 2021 estimated the total number of CHB cases, including foreign-born and US-born persons, in the United States may be as high as 2.4 million with a prevalence of CHB equaling approximately 0.7%.^36^ In 2018, approximately 1649 deaths were reported as caused by HBV.^37^

In the United States, the rate of acute and chronic hepatitis B has declined since the early 1990s following screening of pregnant women and universal vaccination of newborns; it has increased slightly recently with the opioid epidemic.^38^ The reported rate of acute HBV infection has decreased from 8.5 per 100,000 population in 1990 to 0.9 per 100,000 population in 2011.^39^ Between 2000 and 2012, rates declined for all age groups, with the most consistent reduction in HBV incidence among children and adults in their 20s. This corresponds with the generation that began receiving the HBV vaccine as infants.^40^ However, despite the decline in HBV incidence among adolescents and young adults due to vaccination, increases in incidence can be seen in nonurban environments, such as the Appalachian region (Kentucky, Tennessee, and West Virginia), where identified cases reported IVDU as a common risk factor.^41^^,^^42^ Studies have shown CHB to be pervasive in PWIDs with the prevalence of CHB ranging from 3.5% to 20.0% among PWIDs in a variety of settings and 22.6% of PWIDs having evidence of past infection.^43^

In addition, the number of adults with CHB has increased as a result of immigration from endemic countries.^44^ Foreign-born adults are estimated to comprise up to 70% of HBV infections in the US. In a meta-analysis by Kowdley et al, most foreign-born adults with CHB in the US migrated from Asia (58%) followed by Africa (11%) and Central America (7%). Foreign-born individuals who migrated from Africa had the highest average CHB rate (10.3%), followed by foreign-born individuals from Asia (7.27%), Oceania (4.78%), and the Caribbean (4.52%).^30^^,^^45^ Between 1994 and 2003, approximately 40,000–45,000 people entered the US legally from HBV-endemic countries where the prevalence of CHB infection was >2%. In a study of Hmong patients in Minnesota, the HBsAg positivity rate was 18%, with the highest rate of infection among patients 15–19 years of age, where the rate was 28%.^46^^,^^47^ The epidemiology of HBV would be much better defined if HBV testing with the triple panel, which includes HBsAg, antibodies to hepatitis B surface (anti-HBs or HBsAb), and anti-HBc, was performed in all adults and children with risk events or unknown HBV vaccine status.

## Transmission

HBV can be detected in serum, urine, saliva, nasopharyngeal secretions, urine, tears, vaginal secretions, menstrual blood, and semen.^48^ The virus can, therefore, be transmitted by perinatal transmission (perinatal includes up to 4 weeks after birth), horizontal transmission between children in infancy, percutaneous (eg, IVDU) transmission, sexual exposure, or close person-to-person contact in the presence of open cuts and sores (a common transmission method in children).^49^

Perinatal transmission accounts for the majority of HBV transmission worldwide. The probability of becoming chronically infected is inversely correlated to age. The risk of CHB infection is higher when the infection is acquired from an infected mother during the perinatal period due to “tolerance” to the virus as the virus and infant’s immune system interact.^50^ Between 80% and 90% of newborns and children younger than 1 year and 25%–30% of children infected between the ages of 1 and 6 years will develop CHB.^51^ Immunocompetent adults have about a 95% chance of eliminating the virus and remaining protected for life in case of re-exposure.^15^ Based on current CDC guidelines in the *Morbidity and Mortality Weekly Report* 2023, all adults should be tested for HBV with the “triple panel”: HBsAg, anti-HBs, and anti-HBc.^37^

In the US, the most frequent routes of transmission are injection drug use, perinatal, and sexual contact with an infected person. However, in both resource-poor and well-resourced settings, healthcare-associated infection continues to be a significant concern.^52^ Approximately 0.3% of the general population is estimated to be chronically infected with HBV. From 2008 to 2014, 23 acute hepatitis B outbreaks occurred in 175 cases and more than 10,700 persons were notified for screening. Of the 23 outbreaks, 17 outbreaks occurred in long-term care facilities, where most were caused by infection-control lapses during monitoring of blood glucose levels.^53^ Incomplete vaccination of staff, failure to apply universal precautions, and incorrect needle-disposal techniques are the most common reasons for transmission.

## Genotype

The genetic variability of the HBV virus is largely attributed to a lack of proofreading mechanism in the HBV reverse transcriptase responsible for replication of the HBV genomic DNA. HBV has a high mutation rate, with an estimated nucleotide replacement rate of 1.4–3.2 × 10^−5^ per site per year.^54^ This results in frequent errors in nucleotide substitutions during viral replication contributing to genotype, subgenotype, and quasispecies diversity.^54^

HBV genotypes and sub-genotypes are identified by a divergence in their DNA base-pair sequences, and each genotype differs by more than 8%, while each sub-genotype differs by 4%–8%.^55^ The specific locations of the nucleotide differences that define the genotypes and sub-genotypes vary and can be based on the nucleotide sequence variability of the S gene or mutations in the precore/core region.^55^^,^^56^

**Figure 1:**
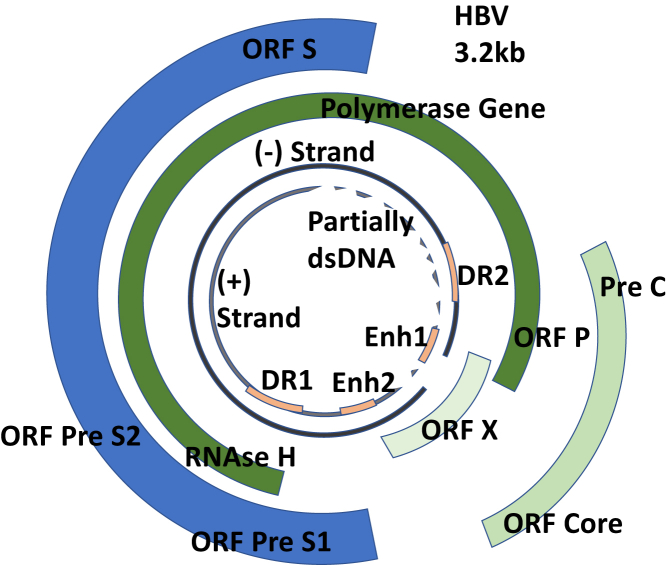
Schematic representation of HBV replication. Library of Science & Medical Illustrations were used in part to create this figure. https://creativecommons.org/licences/by-nc-sa/4.0/. dsDNA, double-stranded DNA; HBV, hepatitis B virus; ORFs, open reading frames.

HBV is a partially double-stranded, relaxed-circular DNA (rcDNA) genome about 3.2 kilobases in length.^54^ The HBV genome consists of 4 partially overlapping open reading frames: C, S, P, and X (Figure 1). The core (C) gene consists of the precore and core region that encodes the HBV e antigen and core protein. The S gene encodes for HBsAg and is divided into 3 sections: pre-S1, pre-S2, and S. The polymerase (P) gene overlaps the S gene and encodes for the HBV-DNA P. The X gene encodes the hepatitis B x antigen that is involved with gene expression, immunologic evasion, and development of chronic liver disease and HCC.^57^

Evidence suggests that the various genotypes and subgenotypes of HBV influence the epidemiology, natural history, and outcomes of the infection.^58^ HBV has 10 different genotypes (A-J) and 40 subtypes, each showing different geographical distributions.^54^ The geographical distribution of HBV genotypes are generally localized by continent with some variation and are summarized in Table 1.^54^

**Table 1:** Global Distribution of HBV Genotypes A-J

Genotype	Distribution^54^
A	Most common in Europe, North America, and some parts of southeastern Africa. It is also found in central and South America, but at a lower frequency.
B	Most common in Asia, particularly China, Taiwan, and Southeast Asia
C	Most common in Asia, particularly Japan, Korea, and China. It is the main genotype in South Korea
D	Found globally, but it’s mostly predominant in the Mediterranean basin, Middle East, and parts of South Asia
E	Primarily in West Africa and central Africa
F	South and central America, particularly Mexico; Thought to be the predominant genotype compared to A and D
G	Found in United States and Europe, and some parts of Asia and Africa
H	Found in central and South America, particularly Mexico and Guatemala
I	Primarily in Southeast Asia, particularly Laos and Vietnam
J	Ryuku Islands of Japan^59^

Current population studies have tried to establish a pattern between HBV genotypes, viral load, and naturally occurring HBV mutations to determine any influence on viral pathogenesis. However, the precise role of viral genotypes in HBV progression has yet to be established. There have been several documented population studies that have suggested that specific viral genotypes can lead to diverse clinical manifestations, including acute hepatitis, chronic hepatitis, liver cirrhosis, and HCC.^55^ For example, chronic infection with genotypes C and D has been associated with a higher risk of advanced liver disease and HCC.^60^, ^61^, ^62^, ^63^, ^64^ However, these genotypes are also highly endemic in areas with high perinatal transmission, which can lead to the worsening progression of disease by increasing the risk and duration of CHB infection. Perinatal transmission can also result in higher viral loads and promote immune tolerance to the virus, further contributing to disease progression.^65^ Additionally, individuals infected with HBV at birth or during early childhood are at a particularly high risk of developing HCC due to their longer duration of exposure to the virus compared to those infected later in life. Some authors have suggested that the analysis of genotype representation on the global scale can help predict the risk of liver-related morbidity and guide therapeutic efficacy.^54^^,^^66^ However, current HBV therapy is targeted at stopping viral replication and research has not shown a benefit to targeting viral suppression according to genotype.^49^ With the current medications, viral replication is stopped regardless of genotype. Therefore, genotyping is not performed routinely as, currently it does not impact treatment decisions.

## Viral Replication

Human HBV is a DNA virus belonging to the hepadnaviridae family, which includes a group of hepatotropic DNA viruses that are species-specific and comprise 2 main genera. The orthohepadnavirus genus infects mammals such as woodchucks, ground squirrels, bats, and primates and shares a 70% nucleotide homology. The avihepadnavirus genus infects birds such as ducks, herons, storks, geese, and parrots and shares 80% nucleotide homology.^67^ The homology between the 2 genera is about 40%, sharing a common genomic organization.

HBV comprises an external lipid envelope measuring approximately 42 nm in diameter and an internal icosahedral nucleocapsid measuring approximately 27 nm in diameter. The icosahedral nucleocapsid encloses a partially double-stranded, rcDNA genome made up of approximately 3.2 kilobases.^68^ The virus can survive outside the human body for up to 7 days or much longer in special situations where the virus is protected from desiccation or chemical exposures.

**Figure 2:**
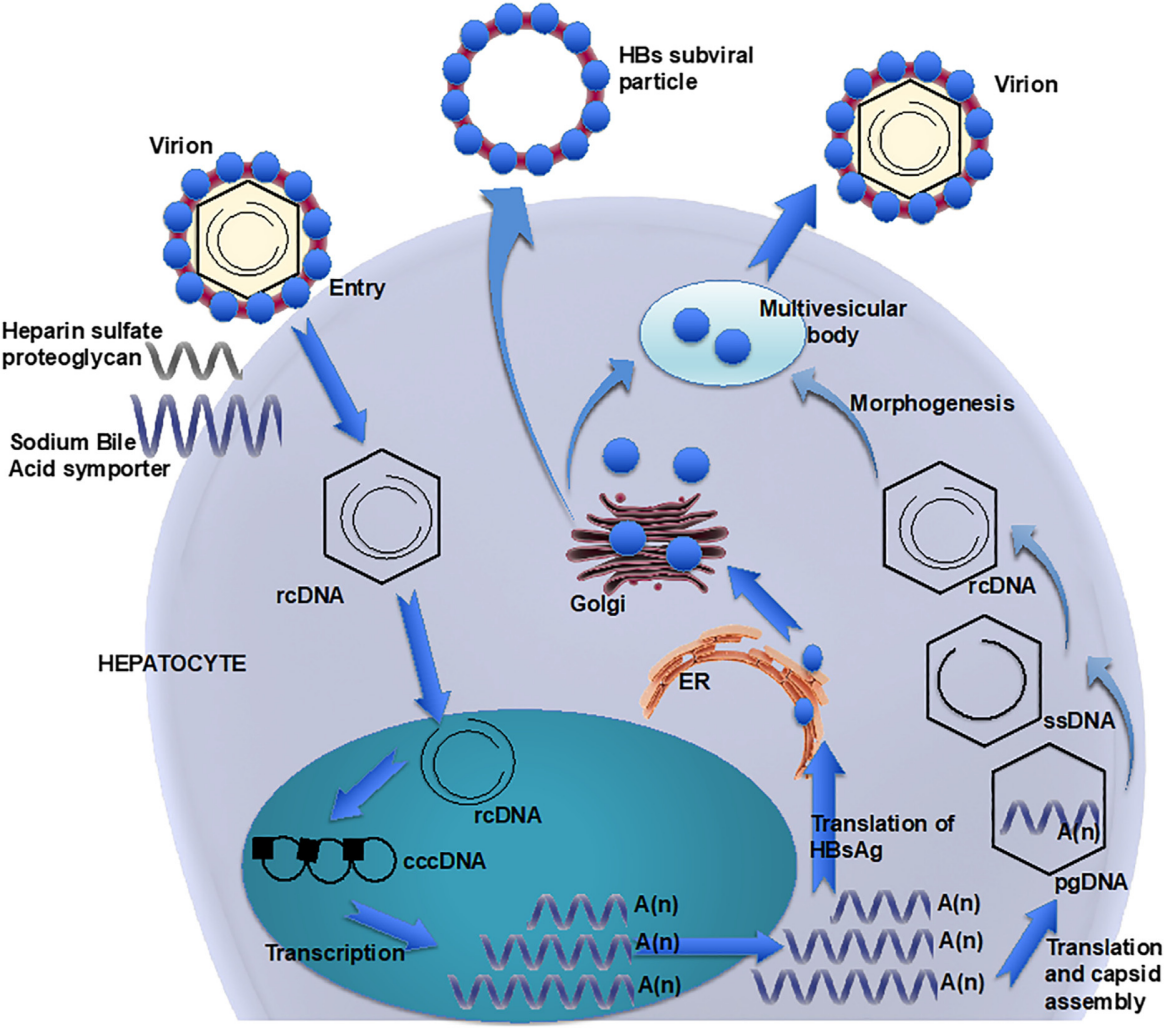
Schematic representation of HBV replication. Library of Science & Medical Illustrations were used in part to create this figure. https://creativecommons.org/licences/by-nc-sa/4.0/. cccDNA, covalently closed circular DNA; ER, endoplasmic reticulum; HBsAg, hepatitis B surface antigen; pgDNA, pre-genomic DNA; rcDNA, relaxed-circular DNA; ssDNA, single-stranded DNA.

The steps involved in HBV replication (Figure 2).

1. Entry into host cells: HBV attaches to hepatocytes via sodium taurocholate cotransporting polypeptide.^24^ The fusion of the viral envelope and endosomal membranes releases the viral capsid from its envelope and the capsid is translocated into the nucleus.
2. Formation of cccDNA: Once inside the nucleus, the circular, partially double-stranded DNA is converted to double-stranded covalently closed circular DNA (cccDNA) through unidentified host enzymes creating a mini chromosome that serves as the template for viral mRNA synthesis.^24^
3. Transcription and translation: The cccDNA is converted into viral mRNA via the host RNA P, which is then exported to the cytoplasm where it undergoes translation to create viral core and surface proteins.
4. Assembly and envelopment: The core protein assembles around the viral RNA to create nucleocapsids, which are then enveloped by the surface proteins resulting in the mature virion.^26^
5. Reverse transcription and integration: The replication of HBV occurs via a reverse transcriptase. The cccDNA is converted into pre-genomic RNA (pgRNA) and the pgRNA, HBV nucleocapsid, and P proteins are encapsulated in the virus core particle where reverse transcriptase occurs. The pgRNA serves as a template for the synthesis of negative single-stranded linear and then partially double-stranded, rcDNA.^68^ Capsids containing a mature, rcDNA genome are sent back to the nucleus for further cccDNA synthesis, where it serves as a template for further transcription and replication. The maintenance of the cccDNA contributes to the chronicity of the virus.

## Serologic and Nucleic Acid Testing

The course of HBV infection is variable and can transition between multiple phases throughout the course of disease. Serologic and molecular markers are used to detect HBV infection or past exposure (Table 2), monitor clinical course of the infection, and assess response to treatment and/or immunization. The most common serological markers of HBV infection include HBsAg, antibody to hepatitis B surface antigen (anti-HBs), immunoglobulin class M antibodies to hepatitis B core antigen (IgM anti-HBcAg), immunoglobulin class G antibodies to hepatitis B core antigen (IgG anti-HBcAg), total anti-HBcAg (combination of anti-HBc IgM and IgG), and HBV-DNA (Table 3). At least 1 marker is present throughout every phase of infection except in rare cases of occult HBV infection, where all seromarkers are negative except nucleic acid testing in the blood or liver tissue.

**Table 2:** Interpretation of Serologic and Molecular Markers of HBV Infection

HBsAg	Total anti-HBc	IgM anti-HBc	Anti-HBs	HBV-DNA	Interpretation
(−)	(−)	(−)	(−)	(−)	1. Never infected 2. Not protected
(+)	(−)	(−)	(−)	(+) or (−)	1. Early acute infection 2. Occurring shortly (up to 18 d) following vaccination, transient
(+)	(+)	(+)	(−)	(+)	Acute infection
(−)	(+)	(+)	(+) or (−)	(+) or (−)	Resolving acute infection
(−)	(+)	(−)	(+)	(−)	Immune; Recovery following past infection
(+)	(+)	(−)	(−)	(+)	Chronic infection
(−)	(+)	(−)	(−)	(+) or (−)	False-positive
(−)	(−)	(−)	(+)	(−)	Immune if anti-HBs serum concentration is ≥ 10 mIU per mL after completion of vaccine series; Passive transfer following administration of HBIG

anti-HBc, antibodies to hepatitis C core; anti-HBs, antibodies to hepatitis B; HBIG; hepatitis B immune globulin; HBsAg, hepatitis B surface antigen; IgM anti-HBcAg, immunoglobulin class M antibodies to hepatitis B core antigen.

**Table 3:** Diagnostic Tests Used to Detect, Characterize, and Monitor HBV Infection

Diagnostic test	Clinical significance	Utilization recommendations
HBsAg	Serologic hallmark of HBV infection; detection of acute and CHB infection	Test on initial presentation Screen in all pregnant women Test annually in patients with inactive infection Repeat if concern for seroconversion (eg, immunocompromised patients)
Quantification of HBsAg	Characterizing phase of infection	Determines risk of mother to child transmission
	Detect treatment response, viral reactivation	Differentiates between HBeAg-negative CHB infection from inactive carriers
	Monitor and risk stratify disease progression (eg, HCC)	Determines frequency of surveillance tests/imaging (eg, ALT, elastography) in patients with inactive infection
		Determine withdrawal of NA therapy in patients
		Identify patients who are likely unresponsive to PegIFN therapy
Anti-HBs	Neutralizing antibodies (antibodies to HBsAg)	Initial presentation
	Differentiate between past infection vs acquired immunity (following vaccination)	Loss of HBsAg in patients with HBV infection
Anti-HBc	Detect exposure to HBV	Initial presentation
	Helpful in differentiating acute, chronic, or previous history of HBV infection	
	IgM antibodies useful if in the early convalescence or ‘window period’ of HBV infection	
HBeAg	Indicator of infectivity, viral replication, and viral levels of HBV-DNA (widely replaced by quantitative HBV-DNA assays)	Initial presentation Every 3–6 mo in HBeAg (+) patients on treatment to detect partial immune control of chronic HBV HBV flares (in setting of fluctuating levels of ALT and HBV-DNA)
Anti-HBe ab	Marker of reduction of replicating virus	
HBV-DNA	Quantitative measurement corresponds to the level of viral replication	Initial presentation
	Differentiate between acute, chronic, and recover/resolution phases of HBV infection	Periodic (every 6–12 mo) measurement in untreated patients
	Assesses efficacy of antiviral treatment	More frequent (every 3 mo) in patients receiving treatment with liberation to less frequent testing after HBV-DNA level is undetectable
		Elevated serum ALT
		Anti-HBc (+) patients who are at increased risk for HBV reactivation (eg, immunosuppressive therapy)

ALT, alanine aminotransferase; anti-HBc, antibodies to hepatitis B core; CHB, chronic hepatitis B virus; HBc, hepatitis B core; HBeAg, hepatitis B e antigen; HBsAg, hepatitis B surface antigen; HBV, hepatitis B virus; HCC, hepatocellular carcinoma; NAs, nucleos(t)ide analogues.

### HBsAg and anti-HBs

HBsAg is the serological hallmark of HBV infection and will be positive in patients who are infected with HBV except for those with occult HBV infection or rare HBsAg mutants. HBsAg is typically present in the serum within 1–10 weeks of infection. CHB infection is diagnosed when HBsAg remains positive for at least 6 months.^69^ Quantitative HBsAg levels can be used to assess response to treatment (especially with interferon), predict and monitor disease progression, and risk stratify development of HCC, infectivity, and risk of HBV transmission. Additionally, it helps to decide when to discontinue treatment with nucleotide or nucleoside analogues and PegIFN.^70^^,^^71^ Sustained responders to PegIFN-alpha tend to show a greater HBsAg decline compared to non-responders. Further prospective studies are required to optimize cutoff levels for HBsAg and to predict HBsAg clearance with special attention to HBeAg seropositivity and HBV genotype. At this time, the 100 IU stopping rule for Asians and the 1000 IU stopping rule for Caucasians is useful especially for providers considering when to discontinue nucleos(t)ide analogues (NAs). On the other hand, decline of HBsAg during NAs is rare and the decline of HBsAg is more apparent in HBeAg-positive patients compared to HBeAg-negative patients.

### HBe and anti-HBe

HBeAg can be present in acute or CHB infection and is an indicator of infectivity, viral replication, and viral levels of HBV-DNA. The core gene gives rise to 2 products called precore (preC) protein and the core protein (referred to as “c” or HBcAg) using the same, and largest (3.5kb) mRNA. PreC is inserted into the endoplasmic reticulum where it is post-translationally modified by cleavage of the amino and carboxyl termini to give rise to HBeAg, which is expressed in hepatocyte cytosol and secreted in its soluble form in the serum.^72^ Mutations occurring at the basic core promoter, which modulate HBeAg at the transcriptional level, and the preC region, which blocks HBeAg production at the translational level, have been identified and result in HBeAg defective viruses. Loss of HBeAg, an immunotoleragen, may lead to greater ability of HBV to evade an immune response, creating a virus with reduced replication and transmissibility but increased virulence.^73^ Interestingly, genotype can affect frequency of mutations leading to a varied expression of HBeAg. For example, a G1896A mutation, which leads to a translational stop codon in the leader sequence of HBeAg protein resulting in the inhibition of protein synthesis, was discovered following studies performed in HBeAg-negative, anti-HBe-positive patients from the Mediterranean.^74^ Therefore, HBeAg-negative/anti-Hbe-positive CHB is the most common form of CHB in Southern Europe and Asia, where 30%–80% of patients with CHB are HBeAg-negative as compared with Northern Europe and the United States where only 10%–40% lack HBeAg.^75^ In this population, a combination of other molecular and serological assays is imperative to increase the diagnostic accuracy and ensure close monitoring in HBeAg-negative, anti-Hbe-positive carriers.

### HBcrAg

Hepatitis B core-related antigen (HBcrAg) is a combination biomarker composed of several antigens expressed from the preC/core gene: HBcAg, HBeAg, and pre-c22 precursor protein.^76^ It has been shown to correlate with intrahepatic cccDNA and HBV-DNA, serum HBV-DNA (regardless of HBeAg status), and to a lesser degree, HBsAg. HBcrAg can be detected in situations where serum HBV-DNA becomes undetectable or HBsAg loss is attained.^77^ Therefore, it may help define the phase of CHB, especially in HBeAg-negative patients, as well as predict HBeAg seroconversion, response to NA or PegIFN-alpha-based treatments, and the risk of HBV reactivation in occult HBV infection.^70^^,^^77^ HBcrAg has been shown to be associated with HCC development in treatment-naïve and treatment-experienced patients as well as prediction of HCC recurrence in postsurgical and post-transplant patients.^76^ Further studies are needed to provide additional evidence of how to best use this marker along with or over other traditional markers, such as HBsAg or HBV-DNA levels in clinical practice. HBcrAg, in conjunction with quantitative HBsAg, can also help establish criteria on whether to continue or discontinue NAs.

## Immunotolerance

Immunotolerance is an outdated term and is being replaced by other terms such as chronic infection with high replication, low inflammation, or simply HBeAg+ “chronic infection.” “Immunotolerance” in patients with CHB was formerly described as HBeAg positivity, high viral replication often with serum HBV-DNA levels >10^6-7^ IU/ ml, persistently normal ALT, and no or minimal evidence of liver disease on histopathology. The mechanism of “immunotolerance” is not yet fully understood but is a state of immune recognition with low rates of inflammation, not immune tolerance. One explanation can be due to ineffective antigen processing and transport to major histocompatibility complex I receptors leading to HBV-specific T cell hypo-responsiveness.^78^ In addition, HBV is able to remain undetected and spread as it is assumed that the adaptive immune response plays a significant role in the pathogenesis of liver disease or viral clearance rather than the innate immune response.^79^ HBeAg may be an immune tolerogen and promote transition to CHB. The innate immune response during the initial phase of infection is modulated by the interaction between HBeAg and toll-like receptor domains leading to suppression of signaling cascades and downregulation of downstream inflammatory factors such as nuclear factor kappa B.^80^ Patients who develop fulminant HBV often harbor variants with a preCmutation that does not allow them to produce HBeAg.^56^ In addition, the offspring of HBeAg-negative HBV carriers have a high probability of developing acute hepatitis but not chronic infection.^81^

Histological evidence of disease is more frequently observed in adults 30–40 years of age. Several studies have shown no or minimal liver disease on serial liver biopsies in patients who remain “immunotolerant” and with normal ALT values.^82^^,^^83^ On the other hand, Kumar et al observed up to 40% of HBeAg-positive patients with persistently normal ALT had significant fibrosis (≥F2, Metavir score) with fibrosis significantly associated with older age.^84^ Results of this study were criticized for a potential selection bias and inconsistent follow-up.^85^

The risk of development of HCC is elevated in the setting of high HBV replication and significant histopathological evidence of disease, which is more commonly observed in older patients, particularly males (age >30–40 years).^70^^,^^86^, ^87^, ^88^ Recent studies have shown patients with immune-tolerant HBV develop HCC at a higher rate compared to patients with immune-active HBV, with a 10-year estimated cumulative incidence of HCC of 12.7% (vs 6.1% in immune-active patients, *P* = .001).^89^ The mechanism of hepatocarcinogenesis in “immune-tolerant” HBV as well as all other phases of disease is clearly linked with a direct relationship to HBV integration, including the number of integrations and location of integrations. Hepatocarcinogenesis is hypothesized to occur in the setting of high viral DNA production, predisposing to higher chances of viral DNA integration into chromosomes and inducing genomic instability.^90^

Monitoring and treatment of “immunotolerant” patients is debatable and is often individualized according to age and histopathological severity, although expansion to treat all or almost all patients is emerging as the standard of care.^91^ The reason against the treatment of HBeAg-positive patients with CHB include low rate of HBeAg clearance, low rate of viral suppression, indefinite treatment duration, possibility of resistance, and lack of evidence showing effect of treatment on a clinical course of disease.^70^^,^^87^ On the other hand, the oncogenic potential of high HBV viral loads, the ability to achieve viral suppression, and the difficulty in recognizing disease phase transition are a few claims that support treatment in this specific population of patients with CHB.^90^ Guidelines for monitoring and treatment of patients in the immunotolerant phase of HBV infection are shown in Table 4. Other approaches suggesting early treatment of “immunotolerant” patients have been proposed.^92^^,^^93^ Particular subgroups of “immunotolerant” patients, such as patients who will receive immunosuppressive treatment or chemotherapy, women with serum HBV-DNA > 10^6-7^ IU/ml during the last trimester of pregnancy, and certain health care professionals with high viraemia levels, are more likely to require or undergo early treatment with anti-HBV agents.^90^

**Table 4:** Guidelines for Monitoring Patients in the Chronic Infection (Formerly Known as “Immunotolerant” Phase)

Society	Guidelines	Evidence/Grade
AASLD	Does not recommend treatment Monitor via measurement of ALT levels every 6 mo for potential phase transition^87^	
APASL	Does not recommend treatment Monitor fibrosis and progression of liver disease via noninvasive measures every 3 mo and proceed to liver biopsy if noninvasive tests suggest fibrosis or if patient has a family history of HCC or cirrhosis^86^	
EASL	Recommends treatment of patients >30 y of age regardless of histology^70^	Evidence III, Grade of recommendation – GR 2
	Untreated patients are recommended to be monitored every 3–6 mo to assess for risk of transmission, reactivation, and HCC^70^	Evidence II, GR 1

AASLD, American Association for the Study of Liver Diseases; ALT, alanine aminotransferase; APASL, Asian Pacific Association for the Study of the Liver; EASL, European Association for the Study of the Liver; HCC, hepatocellular carcinoma.

## Reactivation

Hepatitis B virus reactivation (HBVr) is the loss of immune control in patients with CHB or immunocompromised/immunosuppressed patients with resolved HBV. Immunosuppression implicated in HBVr includes treatments for malignancy, inflammatory bowel disease, rheumatologic diseases, hepatitis C virus treatment with direct-acting antivirals, or stem cell transplantation.^87^ Pathophysiologically, the main driving factor of HBVr is the cccDNA. During an active infection, partial double-stranded DNA is imported to the nucleus of hepatocytes and eventually leads to the formation of cccDNA.^94^ Persistent cccDNA always puts patients at risk for HBVr. HBVr has diverse manifestations, including elevated viral load without clinical signs of hepatitis described as silent activation; clinical/biochemical/histological evidence of hepatitis (HBV hepatitis); or hepatic synthetic dysfunction with encephalopathy and coagulopathy described as (fulminant) liver failure, which can result in liver transplantation or death.^95^

Prevention includes screening patients undergoing immunosuppression, correct risk stratification of HBVr based on screening results, and proper management with antiviral treatment or prophylaxis.^95^ Many guidelines emphasize the importance of screening for HBsAg and anti-HBc in all patients.^70^^,^^86^^,^^87^ As expected, patients with CHB have a higher risk of HBVr than those with resolved infections. Of the immunosuppressive therapies or immunosuppressed patients, the highest risk includes those undergoing B-cell-directed therapies and hematopoietic stem cell transplantation.^95^ Patients undergoing anti-TNF and steroid therapy are at increased risk of HBVr. Patients treated with TNF-a inhibitors had a pooled incidence of HBVr of 4.2% with a higher rate of reactivation in HBsAg-positive patients (15.4%) compared to those with resolved HBV infection (3.0%).^96^ A meta-analysis corroborated these results by showing the pooled rate of HBVr without antiviral prophylaxis was 15.6% in patients with CHB who were treated with TNF-a inhibitors. However, in patients with resolved HBV, the pooled rate of HBVr without antiviral prophylaxis in patients who were treated with TNF-a inhibitors and non-TNF-targeted biologics were 1.4% and 6.1%, respectively.^97^ Long-term biologic therapy using TNF-a inhibitors in patients with resolved HBV infection was not associated with increased risk of reactivation.^98^^,^^99^ Patients on a higher dose of corticosteroids (defined >20 mg/day prednisolone or equivalent) were shown to have a higher risk of reactivation (14.0%) compared to those receiving low-dose systemic corticosteroids (4.5%)^100^ even after prolonged use.^101^^,^^102^

In patients with CHB at risk of reactivation, antiviral prophylaxis should be started prior to immunosuppression and continued even after discontinuation of immunosuppressive agents. Antiviral prophylaxis is generally continued up to 18 months after discontinuation of high-potency therapy due to the increased risk.^86^^,^^103^ The American Association for the Study of Liver Diseases and European Association for the Study of the Liver recommend that high-risk patients should continue to be screened for HBVr after prophylaxis is withdrawn for up to 12 months.^70^^,^^86^^,^^87^ Additionally, the American Association for the Study of Liver Diseases and European Association for the Study of the Liver recommend checking ALT, HBV-DNA, and HBsAg at 1- to 3-month intervals up to 1 year after cessation of therapy and resumption of an antiviral if needed.^70^^,^^87^ APASL recommends monitoring HBV-DNA to stratify if patients require prophylaxis or continued monitoring.^86^

## Conclusion

Although the prevalence of HBV infection has decreased in well-resourced, industrialized countries due to preventive screening and vaccination, most of the world’s populations live in high-prevalence regions. CHB remains a significant cause of cirrhosis and liver cancer, with the incidence continuing to rise. The predominant form of transmission of HBV differs based on the population and region. Serologic and nucleic acid-based testing is used to detect the current or history of HBV infection, track course, evaluate response to treatment or vaccine, and detect reactivation of HBV. The evidence-based policy of prevention and screening for HBV in all adults is a result of our current understanding of HBV. Broad-based vaccination of adults will also help with elimination when used with linkage to the care and treatment of all HBV-DNA-positive patients. All of this can improve outcomes for patients by reducing the incidence of HCC, liver failure, liver transplant, other HBV-related cancers, and extrahepatic manifestations, which will improve patient quality of life and decrease infectivity and self-stigma. However, the global burden of HBV requires further study to first improve HBV interventions, including vaccination, testing, and “curative” treatment, and then to achieve elimination.
